# Single-port robotic-assisted laparoscopic synchronous surgery in pediatric patent processus vaginalis

**DOI:** 10.1186/s12893-024-02392-5

**Published:** 2024-04-13

**Authors:** Geng Li, Heyun Gao, Shanzhen Yu, Yunkai Guo, Tao Hu, Yifan Liu, Guowei Du, Guangbin Huang, Wen Zhang

**Affiliations:** Department of Pediatric Surgery, Zhongnan Hospital of Wuhan University, Wuhan, 430071 China

**Keywords:** Pediatric, Inguinal hernia, Patent processus vaginalis, Single-port robotic-assisted laparoscopic surgery, Surgical technique

## Abstract

**Purpose:**

Patent processus vaginalis (PPV) is usually observed in pediatric abdominal surgery; however, robotic single-port surgery in repairing processus vaginalis has not been reported in children. Herein, we present our clinical experiences in single-port robotic surgeries for PPV repair to evaluate both efficacy and safety.

**Methods:**

Retrospective analysis of patients underwent single-port robotic-assisted laparoscopic surgery for genitourinary diseases from May 2020 and May 2023 in our center. Among these patients, 21 children had PPV repaired at the same time. The case characteristics and follow-up data were recorded.

**Results:**

Twenty-one of the 53 children were found to have PPV during genitourinary surgery. The simultaneous treatment of the primary disease and PPV with a single-port robotic-assisted platform was both convenient and safe. There was no significant increase in total operation time, and no excessive intraoperative hemorrhage was observed in any of the operations. There were no complications observed on follow-up.

**Conclusion:**

With a high incidence of PPV in children, a single-port robotic-assisted procedure is feasible and effective if simultaneously performed when addressing a primary abdominal disease.

## Introduction

In the field of pediatric surgery, congenital inguinal hernia is the most common disease [1], which occurs due to a patent processus vaginalis, wherein the intraperitoneal tissue can protrude. According to previously reported data, the incidence of inguinal hernia in children is 0.8–4.4%, and it more frequently occurs in males [2]. Unlike in adults, high ligation of the sac is an effective treatment of inguinal hernia in children, which was first described by Turner in 1912 [3]. With the advent of laparoscopy, high ligation of the sac with a laparoscopy assistant has become the gold standard of treatment, which has aided in the clearer exploration of the contralateral processus vaginalis. A retrospective analysis indicated that contralateral PPV was observed in 2,233 cases out of 5,370 children (41.7%) [4]. However, it is still controversial as to whether the contralateral processus vaginalis should be addressed during the same procedure. Some reports have demonstrated that metachronous contralateral inguinal hernia is observed in open unilateral hernia repair surgery, with a ratio of 5.8–11.6% [5, 6]. Furthermore, although most PPV cases do not cause harm, they still possess the risk of developing into inguinal hernia.

Robotic-assisted surgery has been frequently performed in pediatric patients in recent years, including for the treatment of choledochal cysts, abdominal tumors, ureteropelvic junction obstruction, duplex kidney, dysplastic kidney, ovarian tumors and other conditions [7, 8]. This technique has been proven to be safe and efficient due to its three-dimensional high-definition view and flexible robotic wrist [9]; however, there has been no report on its use for treating inguinal hernia in children. Our study included 53 children who underwent single-port robotic surgery for genitourinary disease in our clinical center, and 21 of the children had the processus vaginalis repaired at the same time. This study discusses the technique details and preliminary results of single-port robotic-assisted surgery on inguinal hernia repair.

## Methods

Retrospective analysis of patients underwent single-port robotic-assisted laparoscopic surgery for genitourinary diseases from May 2020 and May 2023, performed by a single surgeon at Zhongnan Hospital of Wuhan University, Department of Pediatric Surgery. The surgeon has performed large amounts of multi-port robotic-assisted surgeries, also he has completed animal experiment to master the skill of single-port robotic-assisted surgery. The inclusion criteria were (1) age less than 14 years, (2) single-port robotic-assisted surgery. Exclusion criteria were (1) history of hernia surgery, (2) unclear case documentation. Full informed consent was obtained from the parents of these children. The patients underwent high ligation of the processus vaginalis assisted with a single-port robot, and a 0.5- to 2-year follow-up visit was performed. Content of follow-up was whether metachronous inguinal hernia occurred or not. The study was approved by the Medical Ethics Committee of Wuhan University Zhongnan Hospital (2,022,003 K).

## Surgical technique

After induction of anesthesia and endotracheal intubation, each patient was placed into the supine and Trendelenburg positions, after which the hip was properly padded, and a urinary catheter was inserted to evacuate the bladder.

**Fig. 1:**
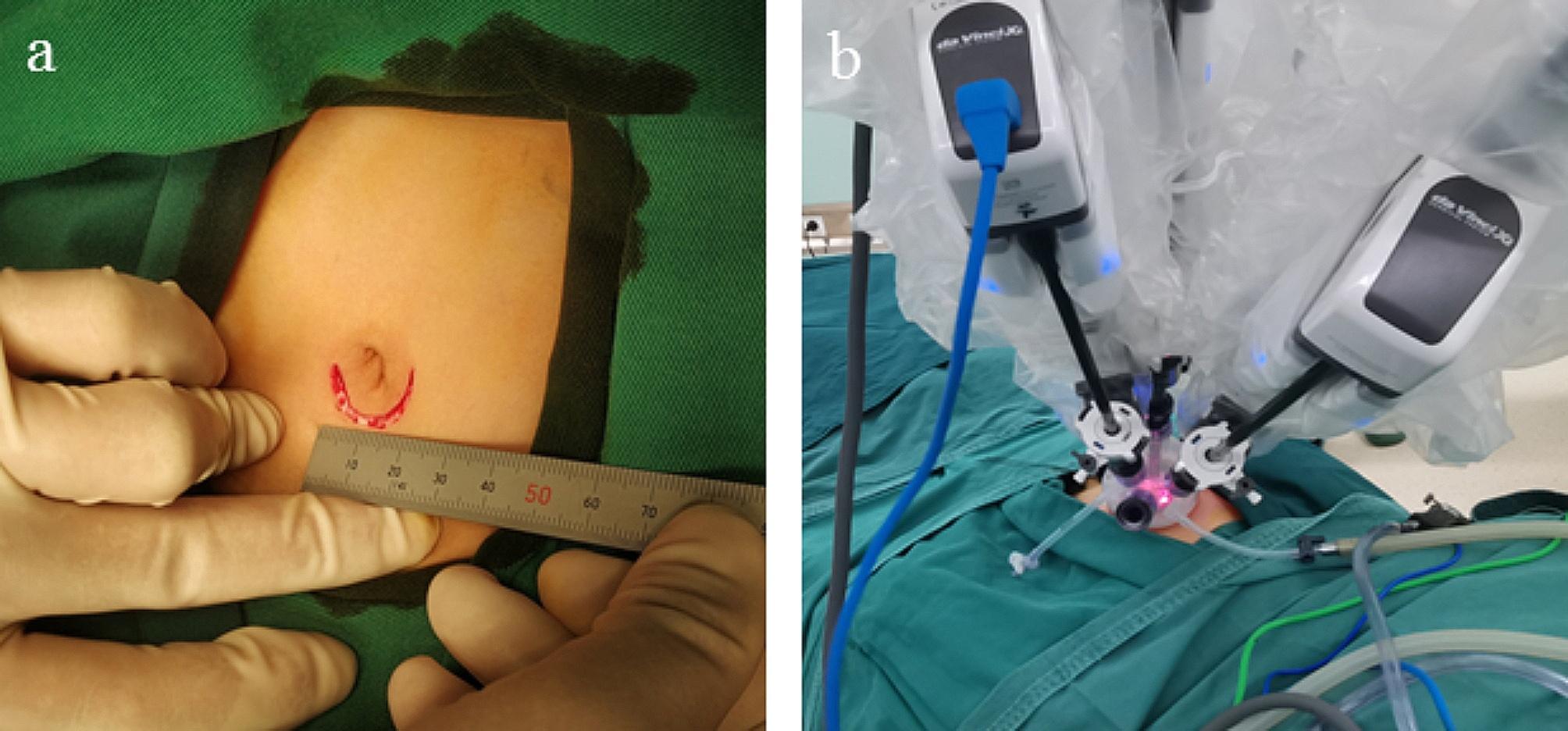
The incision and intraoperative instrument appearance of single-port robotic assistant surgery. **a** A 25 mm arched incision was held along the edge of the umbilical. **b** The appearance of the robot manipulator during the operation

**Fig. 2:**
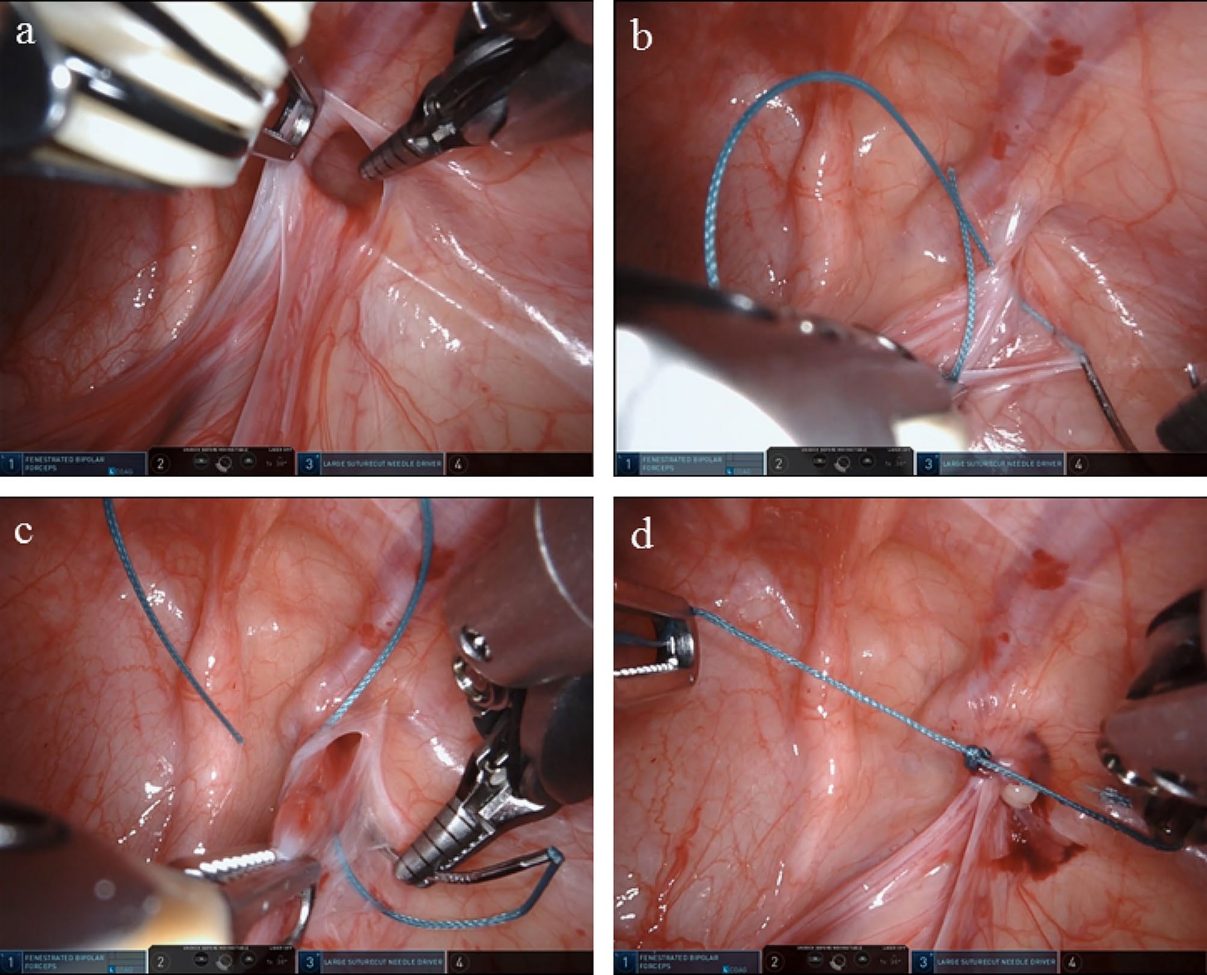
Surgical procedure of patent processus vaginalis high ligation via single-port robotic assistant platform. **a** Intraoperative exploration of the processus vaginalis. **b, c** Purse string suture of patent processus vaginalis. **d.** The appearance of the internal ring after ligation

A 25 mm arched incision was placed along the edge of the umbilicus for the placement of the single port (Fig. 1a). Before docking, a laparoscopic lens was inserted to confirm the presence of a PPV. The lens direction was oriented downward, after which the da Vinci Xi surgical robot was docked to the target area after establishing an artificial pneumoperitoneum (Fig. 1b). For ease of operation, the lens direction was adjusted to an upward orientation. Preparatory work was completed along with the placement of the camera lens and operating apparatus. The operating region was located in the pelvic cavity. The specific location of the PPV was also confirmed (Fig. 2a). Moreover, the ipsilateral hypogastric bowel was pushed aside to fully expose the internal ring. Due to the limited surgical area and with the internal ring on the abdominal wall, the robot manipulator adjusted to a proper angle to avoid any interference.

A nonabsorbable 2 − 0 surgical suture thread with a round needle was placed above the internal ring (along with the inner-side peritoneum), after which the needle came out underneath the internal ring. Subsequently, the needle was again inserted at the spot where it had exited, along with the lateral peritoneum, and the needle finally exited at its initial entry point (Fig. 2b and c). Before starting the knot, the hernial sac was ensured to be devoid of air. Four or five thread nodes were needed to avoid short-term postoperative recurrence (Fig. 2d). Furthermore, the stitching direction was adjusted depending on the exact situation. For males, the needle crossed over the vas deferens and spermatic vessels, whereas for females, it made no difference if ligamentum teres uteri was tied; however, it was necessary to avoid the adnexa uteri.

## Results

A total of 53 children underwent single-port robotic-assisted surgery, of which 21 children (39.6%) underwent high ligation of the processus vaginalis (Table 1), and none of the children had symptomatic PPV before surgery. The following primary diseases were identified: thirteen children had hydronephrosis, three children had duplex kidney, two children had ovarian teratoma, one child had renal dysplasia, one child had renal cyst and one child had vesicoureteral reflux. There were eleven males and ten females, with a male-to-female ratio of 1:0.91. Additionally, 15 cases were diagnosed with unilateral patent processus vaginalis, and the remaining cases were bilateral. Among these cases, the cavernous type accounted for 37% of cases, and the fissured type accounted for 63% of the cases. Nine children (43%) were younger than 2-years-old, eleven cases (57%) were older than 2-years-old and the oldest child was 10.46-years-old.

**Table 1:** Characteristics of patients with patent processus vaginalis

	Numbers of patients(*N* = 21)
Age(years)	4.31 ± 3.80
Sex Male Female	11 10
Primary disease Hydronephrosis Duplex kidney Ovarian teratoma Renal dysplasia Renal cyst Vesicoureteral reflux	13 3 2 1 1 1
Side of PPV Left Right Bilaterally	7 8 6
< 2years	9
≥ 2years	12
Metachronous inguinal hernia	0

Continuous variables as mean ± standard deviation (range).

The extended time for the core procedure (PPV suturing) ranged from 5 to 15 min; for the renal procedure, another 15 min was needed for repositioning of the machine. There was no significant increase in total operation time, and no excessive intraoperative hemorrhage was observed in any of the operations. After surgery, there were no observed complaints of pain in the inguinal region or delays in the initiation of ambulation and feeding. In addition, there was no occurrence of wound infection. Follow-up visits after discharge were performed from 0.5 to 2 years, which revealed no recurrence of metachronous inguinal hernia.

## Discussion

Inguinal hernia has a high incidence in children, and a very small proportion of PPV will eventually develop into inguinal hernia, with a ratio of approximately 15:1, as reported by Yanan Li [4]. A systematic review included 32 studies on unilateral pediatric hernia repaired by laparoscopy, which showed a high percentage (38.5%) of contralateral patent processus vaginalis [10]. Another study reported that 41.7% of 103 children who underwent PPV underwent surgery for hypertrophic pyloric stenosis [11]. Moreover, one study found that 58% of 569 pediatric patients had contralateral patent processus vaginalis [12]. In our clinical center, children who underwent single-port robotic-assisted laparoscopic surgery also showed a high occurrence of PPV. Frequently, surgeons repair unilateral inguinal hernia without contralateral exploration for a balance of risks and benefits [13]. Furthermore, minimal reporting in the article demonstrated that PPV should be repaired in the process of other operations. As age advances, we suggest that PPV repair should be simultaneously performed with a major operation, as it would be beneficial for patients. The risk of developing metachronous or incarcerated inguinal hernia should be considered, which may lead to intestinal necrosis and infertility in females [14].

Open operation for high ligation of the hernial sac has been the standard treatment for inguinal hernia in children; however, spermatic vessels may be damaged because of anatomical complexity in the groin area [15, 16]. In the 1990s, the first laparoscopic repair of inguinal hernia was reported by El-Gohary [17]. With medical developments and the increased performance of minimally invasive procedures, high ligation of the hernial sac by laparoscopy has become the gold standard for treatment. Its advantages include a small incision, fast recovery, safety and reduced occurrence of metachronous contralateral inguinal hernia [18, 19].

In the last decade, robot-assisted surgery has rapidly grown in use in the field of pediatric surgery [20, 21]. This procedure offers better visualization and more precise movement; moreover, the learning curve is less than that of a laparoscope, there has a study reported that one surgeon can be proficient in robotic surgical system when he has performed more than 41 surgeries [22], of course, comparing to multi-port robotic-assisted surgery, it needs more experience to master the skill of single-port robotic-assisted surgery, the surgeon in our center can skillfully perform the operation when he has completed 5 single-port robotic-assisted surgeries. Single-port robotic-assisted laparoscopic surgery is gradually being applied in many medical centers due to its concealed incision [23, 24], it also has some limitations, the robot arms have a restricted range of motion, the arms might be prone to collisions, skilled assistant is needed to adjust the direction of the arms at any time.

Previous reports have discussed inguinal hernia repair by using robot-assisted platforms in adults and adolescents [25, 26]; however, the present research is the first study that investigated the utilization of a single-port robotic-assisted platform for pediatric inguinal hernia, which was performed along with a major surgery. Its elaborate actions provide safer handling of the PPV and make it more convenient to complete bilateral processus vaginalis repair via the single-port robot platform. As has been previously mentioned, no extra wounds or painful occurrences were experienced by the children in this study.

## Conclusion

In summary, according to our findings in performing single-port robotic-assisted laparoscope surgery, it is feasible and safe to complete high ligation of PPV during an abdominal operation. Without increasing the risks in children, it can still prevent PPV from developing into inguinal hernia. However, this surgical technique requires a certain amount of skill and training. And further good quality randomized controlled trials are needed to verify the feasibility and safety of this procedure.

## Data Availability

No datasets were generated or analysed during the current study.

## References

[1] Abdulhai S, Glenn IC, Ponsky TA (2017). Inguinal hernia. Clin Perinatol.

[2] Miltenburg DM, Nuchtern JG, Jaksic T, Kozinetiz C, Brandt ML (1998). Laparoscopic evaluation of the pediatric inguinal hernia–a meta-analysis. J Pediatr Surg.

[3] Miller R, Clarke S (2018). Inguinal hernias in babies and children.

[4] Li Y, Wu Y, Wang C, Wang Q, Zhao Y, Ji Y, Xiang B (2019). Incidence of pediatric metachronous contralateral inguinal hernia and the relationship with contralateral patent processus vaginalis. Surg Endosc.

[5] Tackett LD, Breuer CK, Luks FI, Caldamone AA, Breuer JG, Deluca FG, Caesar RE, Efthemiou E, Wesselhoeft CJ (1999). Incidence of contralateral inguinal hernia: a prospective analysis. J Pediatr Surg.

[6] Ikeda H, Suzuki N, Takahashi A, Kuroiwa M, Sakai M, Tsuchida Y (2000). Risk of contralateral manifestation in children with unilateral inguinal hernia: should hernia in children be treated contralaterally?. J Pediatr Surg.

[7] Denning N, Kallis MP, Prince JM (2020). Pediatric robotic surgery. Surg Clin North Am.

[8] Cundy TP, Shetty K, Clark J, Chang TP, Sriskandarajah K, Gattas NE, Najmaldin A, Yang G, Darzi A (2013). The first decade of robotic surgery in children. J Pediatr Surg.

[9] Tomaszewski JJ, Casella DP, Turner RM, Casale P, Ost MC (2012). Pediatric laparoscopic and robot-assisted laparoscopic surgery: technical considerations. J Endourol.

[10] Muensterer OJ, Gianicolo E (2019). Contralateral processus closure to prevent metachronous inguinal hernia: a systematic review. Int J Surg.

[11] Yan XQ, Zheng NN, Xing FZ, Yu L, Lu W, Duan XF, Yang J, Bian HQ (2015). Incidence and concurrent laparoscopic repair of hypertrophic pyloric stenosis and patent processus vaginalis. Chin Med J (Engl).

[12] Ho IG, Ihn K, Koo EJ, Oh JT (2019). A study of contralateral persistent processus vaginalis in laparoscopic hernia repair in children. Hernia.

[13] Zani A, Eaton S, Hoellwarth M, Puri P, Tovar J, Fasching G, Bagolan P, Lukac M, Wijnen R, Kuebler J, Cecchetto G, Rintala R, Pierro A (2014). Management of pediatric inguinal hernias in the era of laparoscopy: results of an international survey. Eur J Pediatr Surg.

[14] Raicevic M, Saxena A (2018). Laparoscopic management of müllerian duct remnants in the paediatric age: evidence and outcome analysis. J Minim Access Surg.

[15] Steigman CK, Sotelo-Avila C, Weber TR (1999). The incidence of spermatic cord structures in inguinal hernia sacs from male children. Am J Surg Pathol.

[16] Dehner LP (1999). Inguinal hernia in the male child: where the latest skirmish line has formed. Am J Surg Pathol.

[17] El-Gohary MA (1997). Laparoscopic ligation of inguinal hernia in girls. Pediatr Endosurgery Innovative Techniques.

[18] Shalaby R, Ismail M, Dorgham A, Hefny K, Alsaied G, Gabr K, Abdelaziz M (2010). Laparoscopic hernia repair in infancy and childhood: evaluation of 2 different techniques. J Pediatr Surg.

[19] Zhong H, Wang F (2014). Contralateral metachronous hernia following negative laparoscopic evaluation for contralateral patent processus vaginalis: a meta-analysis. J Laparoendosc Adv Surg Tech A.

[20] Mittal S, Srinivasan A (2021). Robotics in pediatric urology: evolution and the future. Urol Clin North Am.

[21] Cave J, Clarke S (2018). Paediatric robotic surgery. Ann R Coll Surg Engl.

[22] O’Brien ST, Shukla AR (2012). Transition from open to robotic-assisted pediatric pyeloplasty: a feasibility and outcome study. J Pediatr Urol.

[23] Rosales-Velderrain A, Alkhoury F (2017). Single-port robotic cholecystectomy in pediatric patients: single institution experience. J Laparoendosc Adv Surg Tech A.

[24] Klazura G, Sims T, Rojnica M, Koo N, Lobe T (2021). Single port robotic splenectomy for pyruvate kinase deficiency in a five-year-old patient, a case report of a surgical first. Int J Surg Case Rep.

[25] Bosi HR, Guimarães JR, Cavazzola LT (2016). Robotic assisted single site for bilateral inguinal hernia repair. ABCD Arquivos Brasileiros De Cirurgia Digestiva (São Paulo).

[26] Hey MT, Mayhew MM, Rico S, Calisto J, Alkhoury F (2021). Initial experience with robotic inguinal hernia repair in the adolescent population. J Laparoendosc Adv Surg Tech A.

